# Tau Protein: Function and Pathology

**DOI:** 10.1155/2012/707482

**Published:** 2012-08-15

**Authors:** Hanna Rosenmann, David Blum, Rakez Kayed, Lars M. Ittner

**Affiliations:** ^1^Department of Neurology, The Agnes Ginges Center for Human Neurogenetics, Hadassah Hebrew University Medical Center, Ein Karem, Jerusalem 91120, Israel; ^2^Inserm U837, “Alzheimer & Tauopathies” Jean-Pierre Aubert Research Centre, University Lille-Nord de France, UDSL, Lille, France; ^3^Departments of Neurology and Neuroscience & Cell Biology, George and Cynthia Mitchell Center for Neurodegenerative Disease, University of Texas Medical Branch, Galveston, TX 77555, USA; ^4^Alzheimer's and Parkinson's Disease Laboratory, Brain and Mind Research Institute, University of Sydney, Camperdown, NSW 2050, Australia

In the last decade, the fundamental role of the microtubule-associated protein tau in neurodegeneration and dementia has been widely accepted. The generation of various transgenic models for tau pathology, varying in expressed mutations and driving promoters with either permanent (constitutive) or inducible expression, as well as the use of alternative animal models (fly, zebra fish, and rat) provided tools for studying mechanistic aspects of tau pathology and developing therapeutic approaches. It is now well established that pathological forms of tau (hyperphosphorylated, aggregated, and truncated) are a major cause of dementia, rather than being only a secondary effect to the amyloid pathology in Alzheimer's disease (AD). There is a link between these two AD pathologies, tau and amyloid, with tau pathology being downstream to amyloid pathology, yet tau pathology can develop and respond independently of amyloid plaques. The direct evidence for tau pathology developing independently of amyloid, being sufficient to cause dementia and neurodegeneration, is the fact that there are various diseases with isolated tau pathology (frontotemporal dementia, Pick's disease, etc.), one disease with both pathologies (AD), while there is no dementia disease with isolated amyloid pathology without tau pathology present. These findings supported the concept that amyloid toxicity is tau dependent and that blocking/reducing the pathological effects of tau may be protective against the harmful effects of amyloid pathology, a concept that has indeed proven feasibility in various studies. 

Much evidence has been accumulated pointing to the contribution of tau to AD pathology by two mechanisms: loss of function (such as stabilization of microtubules) as well as gain of toxic function (aggregation and deposition as neurofibrillary tangles). Recently, new concepts emerge contributing to our understanding of the pathogenesis of tau pathology, particularly the identification of toxic soluble oligomers of tau, arguing for these isoforms being the main toxic forms of the taupathology; and the concept that tau pathology may spread in the brain by a prion-like mechanism, possibly involving a transsynaptic mechanism of spreading along anatomically connected networks. 

These accumulating data provide a better understanding of tau pathogenesis, and given the disappointing clinical outcomes of antiamyloid therapeutic approaches, led the scientific community to devote much more effort into studying tau pathology, and into developing tau-targeted therapeutic approaches, such as tau immunotherapy, kinase inhibitors, or microtubule stabilizers.

In this Special issue of International Journal of Alzheimer's Disease, the investigators contributed review articles as well as original research articles that stimulate the continuing efforts towards understanding tau pathology in AD and other tauopathies as well as unravel the physiological functions of tau, in an effort to develop new treatments. 

The paper by the F. Van Leuven's group: “Protein tau: prime cause of synaptic and neuronal degeneration in Alzheimer's disease,” which discusses the relevance of tau in Alzheimer's disease and frontotemporal dementias. This concise and clear review covers the major discoveries and the many still remaining questions in the field.

The L. M. Ittner group contributed an excellent review paper: “Lessons from tau-deficient mice.” This paper takes you through the under-reported part of the tau story; it summarizes the consequences for tau loss of function and its possible role in neurodegeneration. The authors thoroughly reviewed the different tau knockout models and addressed the pathophysiology of various tau models. Tau mediates A*β* toxicity and perhaps the toxicity of other amyloidogenic proteins, hence, the characterization of tau knockout animal models is critical for our understanding of the complex molecular signaling pathways in Alzheimer's Disease.

S. S. Hébert and his colleagues, review in their article: “MicroRNAs and the regulation of tau metabolism” what is known about the transcriptional and posttranscriptional regulation of tau. They discussed clearly the role of micro RNAs (miRNAs) in this process, with a focus on miR-16 and miR-132 as putative endogenous modulators of neuronal tau phosphorylation and tau exon 10 splicing, respectively. They speculated that miRNAs may contribute to sporadic forms of tauopathies.

The review from M. Gistenlinck and collaborators, entitled “*Drosophila *models of tauopathies: what have we learned?” is focused on how and why Drosophila is helpful to modelize Tau pathology. Further, contributors explain why such simple models are important to unravel new pathophysiological hypothesis from genetic screening. Finally, authors describe clearly how Drosophila models are unvaluable tools to reconcile genome wide association studies with pathophysiology. 

The group of Gozes in the paper: “Tau and caspase 3 as targets for neuroprotection” shows clearly that in an in vitro model for ischemia, in primary neuronal cultures subjected to oxygen-glucose deprivation that causes an increase in the levels of active caspase-3 and hyperphosphorylated tau, both processes are prevented by either the NAP peptide or caspase-inhibitor treatment. This group suggests that caspase activation may be an upstream event to tau hyperphosphorylation.

M. Kolarova and colleagues, in their contribution entitled “Structure and pathology of tau protein in Alzheimer disease” give us a comprehensive overview of what tau is and how tau can be modified. This contribution particularly emphasized that posttranductional modifications of Tau are important regulatory steps of its aggregation. Also, authors point out importance of Tau truncation which may additionally play an important role in AD-related pathophysiology. 

The final paper of this special issue entitled: “Tau phosphorylation by GSK3 in different conditions” J. Avila et al. review comprehensively the complex consequences for tau of being a phosphoprotein. Focusing on serine/threonine phosphorylation they discuss that depending on the modified residue in tau molecule, phosphorylation could be protective, like in processes like hibernation, or toxic like in development of tauopathies, characterized by a hyperphosphorylation and aggregation of tau.

*Hanna Rosenmann* *Hanna Rosenmann* *David Blum* *David Blum* *Rakez Kayed* *Rakez Kayed* *Lars M. Ittner* *Lars M. Ittner*

